# Establishment of a prognostic scoring model for regional recurrent nasopharyngeal carcinoma after neck dissection

**DOI:** 10.20892/j.issn.2095-3941.2019.0263

**Published:** 2020-02-15

**Authors:** Xiaoyun Li, Chao Lin, Jinjie Yan, Qiuyan Chen, Xuesong Sun, Sailan Liu, Shanshan Guo, Liting Liu, Haojun Xie, Qingnan Tang, Yujing Liang, Ling Guo, Hao Li, Xuekui Liu, Xiang Guo, Linquan Tang, Haiqiang Mai

**Affiliations:** ^1^State Key Laboratory of Oncology in South China; Collaborative Innovation Center for Cancer Medicine; Guangdong Key Laboratory of Nasopharyngeal Carcinoma Diagnosis and Therapy, Sun Yat-sen University Cancer Center, Guangzhou 510060, China; ^2^Department of Nasopharyngeal Carcinoma, Sun Yat-sen University Cancer Center, Guangzhou 510060, China; ^3^Department of Head and Neck Surgery, Sun Yat-sen University Cancer Center, Guangzhou 510060, China

**Keywords:** Nasopharyngeal carcinoma, recurrence, lymphatic metastasis, operation, prognosis

## Abstract

**Objective:** The main aim of this study was to establish a scoring model to predict risk of progression and survival in patients with regionally recurrent nasopharyngeal carcinoma (NPC).

**Methods:** Three hundred and forty-eight patients subjected to neck dissection from 2003 to 2017 were included for study. Clinicopathologic information for each patient was analyzed. Independent prognostic factors were selected using the Cox proportional hazards model and incorporated into the scoring model. Concordance index (C-index) and calibration curves were used to verify discrimination and calibration, respectively and the results validated using bootstrap resampling.

**Results:** Microscopic positive lymph node > 2 [hazard ratio (HR), 2.19; 95% confidence interval (CI), 1.30–3.68; *P* = 0.003], extranodal extension (HR, 2.75; 95% CI, 1.69–4.47; *P* < 0.001), and lower neck involvement (HR, 1.78; 95% CI, 1.04–3.04; *P* = 0.034) were identified from multivariate analysis as independent factors for overall survival (OS). A qualitative 4-point scale was generated to stratify patients into 4 risk groups for predicting OS and progression-free survival (PFS). The novel scoring model demonstrated enhanced discrimination (C-index = 0.69; 95% CI, 0.62–0.76) relative to the original recurrent tumor-node-metastasis (rTNM) staging system (C-index = 0.56; 95% CI, 0.50–0.62), and was internally validated with a bootstrap-adjusted C-index of 0.70. The calibration curve showed good agreement between predicted probabilities and actual observations.

**Conclusions:** The scoring system established in this study based on a large regionally recurrent NPC cohort fills a gap regarding assessment of risk and prediction of survival outcomes after neck dissection in this population and could be further applied to identify high-risk patients who may benefit from more aggressive intervention.

## Introduction

Nasopharyngeal carcinoma (NPC) is a unique type of head-and-neck cancer endemic in Southeast Asia, with an annual incidence of 25–50 per 100,000 individuals in high prevalence areas of China^1^. In recent years, advances in imaging techniques, radiotherapy, and chemotherapy have boosted the complete remission rate of non-metastatic NPC and facilitated effective treatment of locally advanced NPC. However, updated data from a meta-analysis^2^ disclosed locoregional failure in 18% NPC patients after radical radiotherapy and regional recurrence in 7%–16% patients^3,4^. Despite the greatly improved survival rates in newly diagnosed patients, prognosis of regionally recurrent NPC is far from satisfactory. The reported 5 year disease-free survival rates range from 26.3% to 41.8% and overall survival (OS) rates from 51.2% to 63.7%^5–7^. With regard to management of this group of patients, there is no consensus on the optimal treatment based on patient risk levels.

The American Joint Committee on Cancer (AJCC) staging system, the principal instrument currently used to predict prognosis of newly diagnosed NPC, does not apply to relapse patients due to its unbalanced distribution in this population along with weakness for risk stratification. Limitations of original elements for determining N status in predicting outcomes, such as lymph node (LN) diameter and laterality, have been reported by a number of studies, highlighting the demand for a more adaptive grading system.

Recent reports suggest that total LN examined, positive LN (PLN) counts, and PLN ratio (PLNR) in neck dissection are significant prognostic factors correlated with oncologic outcomes in head and neck cancers^8–11^. Given that the majority of regional relapse patients were treated principally *via* surgery, the current retrospective cohort study was conducted to elucidate the contributory roles of these factors to recurrent NPC and investigate the effects of their combination with other important prognosticators to establish an improved grading system for estimating the risk of tumor relapse.

## Materials and methods

### Patients

Between January 1, 2003, and December 31, 2017, 455 consecutive NPC patients who underwent neck dissection as salvage surgery in the Head and Neck Surgery Department of the Sun Yat-Sen University Cancer Center were selected. “State of recurrence” was defined as LNs that achieved complete response within 3 months after treatment but reappeared 3 months later. Patients complying with the following inclusion criteria were enrolled: (a) age 18 to 70 years, (b) initially diagnosed with biopsy-proven World Health Organization type II or III NPC, (c) received radical radiotherapy initially with or without chemotherapy, (d) received neck dissection on the first episode of recurrence, and (e) cervical LN recurrence was confirmed by postoperative pathology. Exclusion criteria were as follows: (a) patients experienced regional recurrence with simultaneous local recurrence or distant metastasis, (b) cases of concurrent retropharyngeal LN metastasis, and (c) detection of a second malignancy before or after surgery. All research procedures were approved by the Ethics Committee of the Sun Yat-sen University Cancer Center (Approval No. GZR2017-206). Written informed consent for data collection and analysis was obtained from all patients.

### Treatment

All patients underwent one of the following neck dissection procedures: (a) selective neck dissection (SND), (b) modified radical neck dissection (MRND), and (c) RND. Selection of the appropriate procedure was based on preoperative imaging examinations [computed tomography, magnetic resonance imaging (MRI) or ultrasound] and intraoperative exploration. In cases where isolated LN recurrence or recurrence in less than 3 consecutive levels were identified via imaging tests or intraoperative exploration, SND was implemented. If recurrent LNs were distributed in 3 or >3 levels without obvious infiltration into adjacent tissues or invasion of the spinal accessory nerve, internal jugular vein, and/or sternocleidomastoid muscle verified during intraoperative exploration, MRND was performed to preserve important structures. In all other cases, RND was performed.

Upon detection of obvious perinodal adhesions with unclear boundaries or tumors encasing important nerves, blood vessels or sternocleidomastoid muscle during the operation, patients were recommended adjuvant chemotherapy and/or radiotherapy. For postoperative radiation, accumulated doses of 50 to 60 Gy in 25 to 30 fractions were administered to the neck region. The commonly used adjuvant chemotherapy regimens included docetaxel plus cisplatin, cisplatin plus 5-fluorouracil, and gemcitabine plus cisplatin for 3 or 4 cycles.

### Data collection and assessment

Demographics and clinical information, such as initial tumor-node-metastasis (TNM) stage, previous treatments, rN status, Epstein–Barr virus (EBV) DNA values, and postoperative therapy, were collected. rN recurrence was staged according to the 8th Edition of the Union for International Cancer Control TNM staging system based on preoperative imaging tests. If patients experienced multiple regional relapse, evaluation of the first recurrence was performed. Surgical and pathologic factors, including surgical method, number of LNs examined, PLN count, LNR, LN size, extranodal extension (ENE), pathologically confirmed laterality, and location of PLNs, were evaluated. Results were interpreted independently by two experienced pathologists. ENE was defined as the invasion of neoplastic cells into perinodal fibrillar connective tissue or adipose tissue under a microscope (**Supplementary Figure S1**). Lower neck involvement referred to postoperative pathology-confirmed level IV/Vb invasion. The primary endpoint measures, OS and progression-free survival (PFS), refer to the time interval between salvage surgery and death or progression due to any cause. Patients were censored on the date of last follow-up.

### Statistical analysis

The number of LNs examined, PLNs, and LNR were dichotomized by median value. Baseline characteristics in patients were compared using the Chi-squared (χ^2^) test for categorical variables. Kaplan–Meier curve analysis was used to estimate survival and the log-rank test applied to measure the significance of differences. Univariable and multivariable survival analyses were conducted using the Cox proportional hazards model. A scoring system taking all significant prognostic factors in the multivariable Cox regression model into account was proposed. The predictive performance of the scoring system was evaluated using the concordance index (C-index), which measures the concordance level between the predicted model and actual chance of experiencing the events, and the discrimination ability was compared to the rN stage. Internal validation was performed with the bootstrap-adjusted C-index with 1,000 resamples. A calibration curve was derived to assess the fit of the regression model. All statistical analyses were performed using SPSS version 23.0 (SPSS Inc., Chicago, IL, USA) and R statistics version 3.5.0 (http://www.r-project.org/). A two-tailed *P* < 0.05 was considered statistically significant.

## Results

**Figure 1:**
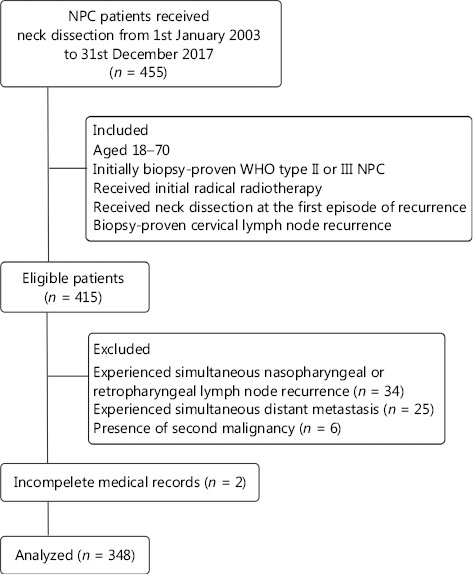
Flow diagram of the study selection process (inclusion and exclusion criteria).

Among the 455 patients previously diagnosed with NPC and subjected to neck dissection between 2003 and 2017, 415 met the inclusion criteria, and after the procedure of elimination, 348 were finally included for analysis (**Figure 1**). Fifty-six (16.1%) of the patients received radical radiotherapy and 257 (73.9%) underwent platinum-based concurrent chemoradiotherapy with or without induction or adjuvant chemotherapy. No significant differences in OS were observed between groups administered initial radiotherapy and chemoradiotherapy (71.4% *vs.* 80.8%; *P* = 0.380). The median [interquartile range (IQR)] interval to recurrence was 20.2 (12.2–39.4) months and median (IQR) follow-up time was 30.8 (17.2–46.7) months. The 3-year OS rate was 79.3% [95% confidence interval (95% CI), 78.4%–87.8%]. In total, 72 patients died (of any cause). One hundred and thirty patients experienced tumor progression after surgery, which accounted for 86.1% deaths. Thirty-six cases (27.7%) of progression were ascribed to distant metastasis and 94 (72.3%) to locoregional recurrence. Seventy-five (21.6%) patients experienced subsequent or repeated cervical recurrence. Among these patients, 55 (73.3%) had *in situ* recurrent disease and their OS rates were significantly poorer than those showing relapse elsewhere (54.3%; 95% CI, 39.4%–69.2% *vs.* 80.8%; 95% CI, 61.2%–100.0%; *P* = 0.03).

The average ± SD number of total LNs examined was 9.7 ± 7.3 and mean ± SD number of PLNs was 3.2 ± 3.9, with median PLNR of 0.33. Relevant demographic and clinicopathologic features of patients (**Table 1)** were stratified into groups of PLN ≤ 2 and > 2. A higher proportion of male patients was recorded in the cohort of PLN > 2 (*P* = 0.029). Initial T stage and total stage were comparable between the two groups whereas advanced N stage was associated with more recurrent PLNs to a near-significant extent (*P* = 0.058). Patients in the PLN > 2 group additionally presented with a significant higher percentage of bilateral invasion (1.4% *vs.* 9.5%; *P* < 0.001), lower neck invasion (11.8% *vs.* 41.0%; *P* < 0.001), and postoperative adjuvant therapy (21.3% *vs.* 36.5%; *P* = 0.002). Other variables were comparable between the two groups.

**Figure 2:**
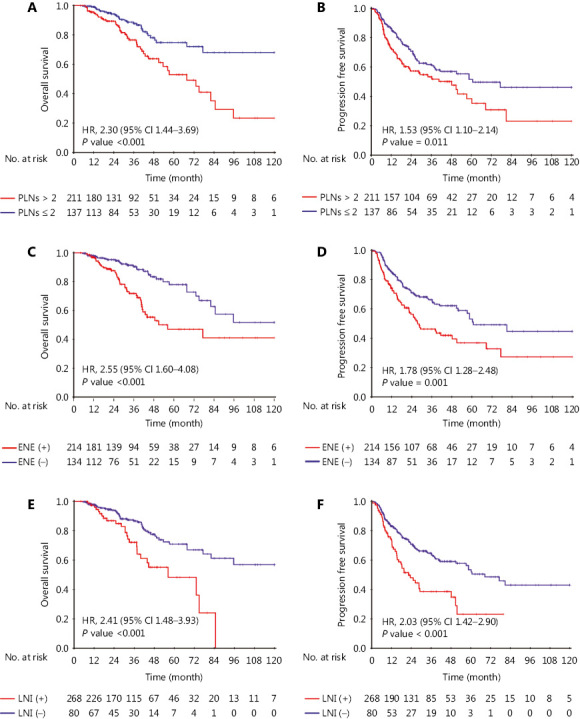
Kaplan–Meier curves for overall survival (OS) and progression-free survival (PFS) stratified by number of positive lymph nodes (PLNs) (A, B), extranodal extension (ENE) (C, D), and lower neck involvement (E, F).

In univariable analysis (**Table 2**), total LN number did not demonstrate predictive value for any endpoint while PLN > 2 was strongly correlated with poorer 3-year OS (75.0%; 95% CI, 66.2%–83.8% *vs.* 87.7%; 95% CI, 82.2%–93.2%; *P* < 0.001) and 3-year PFS (51.7%; 95% CI, 42.1%–61.3% *vs.* 60.8%; 95% CI, 53.2%–68.4%, *P* < 0.001), with the same applying for LNR > 0.33 (OS: 79.6%; 95% CI, 72.5%–86.7% *vs.* 87.1%; 95% CI, 80.8%–93.4%; *P* = 0.027; PFS: 52.0%; 95% CI, 43.8%–60.2% *vs.* 88.3%; 95% CI, 83.4%–93.2%; *P* = 0.005). Tumor characteristics, such as ENE and lower neck invasion, were significant prognostic factors for OS (**Figure 2**). In a multivariate model adjusted for relevant confounders, PLN number [hazard ratio (HR), 2.19; 95% CI, 1.30–3.68; *P* = 0.003], ENE (HR, 2.75; 95% CI, 1.69–4.47; *P* < 0.001) and lower neck involvement (HR, 1.78; 95% CI, 1.04–3.04; *P* = 0.034) remained independent risk factors for poorer OS. Similar results were obtained for PFS. LNR was removed from the model on account of the multicollinearity existing between PLNs and LNR. Both LN size and bilateral invasion failed to achieve significance as predictors of survival in multivariable analysis.

**Figure 3:**
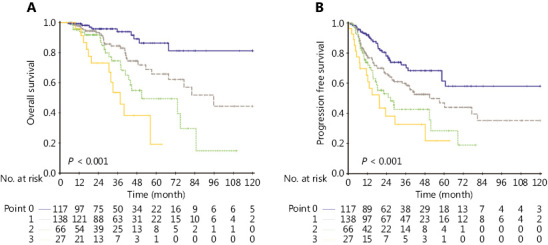
Kaplan–Meier curves for overall survival (OS) and progression-free survival (PFS) in different risk groups based on the novel scoring model.

**Figure 4:**
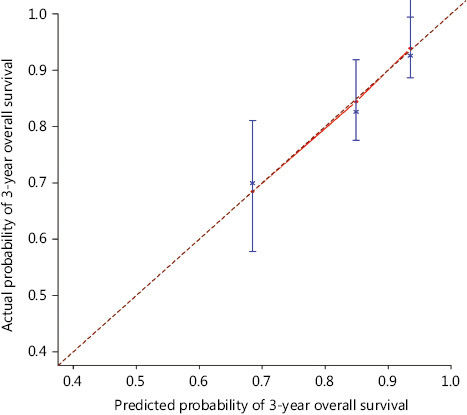
Model calibration curve showing the predicted and actual probability of overall survival (OS) at 3 years. Vertical bars represent 95% confidence intervals (CI).

Given the high prognostic value of PLNs, ENE and lower neck involvement, we devised a qualitative 4-point scale to assess hazards as well as predict progression and survival. Points 0 to 3 corresponded to the following 4 groups: low risk, moderate risk, moderately high risk, and high risk. Patients with PLN number ≤ 2, ENE (−), and without lower neck invasion were scored 0. Points 1 or 2 were assigned in cases where any one or two of the following conditions were satisfied: PLN > 2, ENE (+), and lower neck involvement (+). Patients positive for all conditions were scored 3. OS and PFS were assessed based on the score (**Table 3**). The estimated 3-year OS rates were 92.3%, 79.0%, 66.7%, and 55.6%, while PFS rates were 74.4%, 57.2%, 47.0%, and 37.0% for patients in the low risk, moderate risk, moderately high risk, and high risk groups, respectively (**Figure 3**). The novel scoring model demonstrated significantly enhanced discriminative competence (C-index = 0.69; 95% CI, 0.62–0.76) relative to the original rN stage (C-index = 0.56; 95% CI, 0.50–0.62; *P* < 0.001). Furthermore, in subgroup analysis stratified by SND and RND, the scoring model retained good discrimination ability (C index for SND cohort = 0.68; 95% CI, 0.58–0.78; C index for RND cohort = 0.70; 95% CI, 0.59–0.81). The survival curves for OS in SND and RND groups are shown in **Supplementary Figure S2**. With regard to internal validation, the bootstrap method revealed a C-index of 0.70 (95% CI, 0.62–0.78) for the scoring model and 0.54 (95% CI, 0.48–0.60) for rN stage, indicating satisfactory concordance and discrimination of the scoring method and enhanced potential to assess risks and predict outcomes. The calibration curve validated the concurrence between the predicted and actual observations (**Figure 4**).

In patients with preoperative plasma EBV DNA results, EBV DNA ≥ 1500 was a significant prognosticator for both OS (72.7% *vs.* 89.3%; HR, 2.25; 95% CI, 1.26–4.02; *P* = 0.005) and PFS (46.9% *vs.* 71.7%; HR, 2.04; 95% CI, 1.40–2.97; *P* < 0.001). However, as a strong correlation existed between the preoperative EBV DNA level and lower neck involvement (*P* = 0.003), it failed to serve as an independent risk factor in multivariable analysis (*P* = 0.06). Overall, 96 (27.6%) patients received adjuvant therapy, including chemotherapy and/or radiotherapy, after dissection. As postoperative treatment was only performed in patients with extensive infiltration of tumors that could not be fully eradicated by dissection, prognosis in these cases was unsatisfactory. The addition of adjuvant therapy failed to provide survival benefits in all risk groups and survival outcomes remained poorer in these patients, compared to those subjected to dissection alone (OS, 71.9% *vs.* 82.1%; *P* = 0.01; PFS, 55.2% *vs.* 61.1%; *P* = 0.250).

## Discussion

This large cohort study on patients with regionally recurrent NPC offers valuable insights into improving treatment of a regional recurrence-only population, and provides concrete survival outcomes. Based on the results, we have devised a scoring model for risk stratification, which greatly outperforms the TNM staging system. Undifferentiated or poorly differentiated NPC is distinguished from other head and neck tumors based on excellent radiosensitivity, usually with achievement of complete remission after initial radiotherapy. However, recurrent NPC does not respond well to re-irradiation owing to high vulnerability of normal tissues to the effects of irradiation and the existence of radioresistant tumor cells. In these cases, surgery is administered empirically to patients with regional recurrence. Due to the rarity of this disease, few studies have provided comprehensive information on the risk factors and prognosis for this population. Consequently, no consensus has been achieved on how to stratify patients and manage treatment. The AJCC staging system is not applicable as many nodal recurrences appear isolated. The bilateral invasion rate in our study was 9.4% according to preoperative imaging tests whereas only 4.7% was confirmed by pathology and 10.8% and 10.5% were reported in studies by You et al.^12^ and Chan and Chan^13^, respectively. Moreover, similar survival outcomes between rN1 and rN2 patients signified poor discrimination of the recurrent TNM (rTNM) system. You et al.^12^ proposed a staging system for recurrent NPC, classified nodal recurrence into resectable and unresectable groups, and subsequently regrouped patients with the combination of local recurrence. The survival results of a regional recurrence-only population were not assessed and owing to a range of subgroups, the staging system was complex and difficult to apply. Here, we have identified potential risk factors in nodal recurrent NPC patients and established a simple postoperative scoring model to precisely predict prognosis and guide subsequent treatment.

Total LN count, PLN number, and LNR are three closely associated factors reported by different studies as strong predictors of recurrence and survival in head and neck cancers^11,14–17^, prostate cancer^18,19^, and breast cancer^20^. In our analysis, total LN examined showed no correlation with outcomes as the value was largely determined by the type and extent of neck dissection. As 69.5% recurrent NPC patients received SND, there was a lower likelihood of harvesting as many LNs as with RND. While LNR was also an important prognostic factor in head and neck cancers, a direct correlation between LNR and extent of dissection inherently existed. The cutoff point of LNR was 33% in our study, and varied from 6% to 16% in other reports^15,21–23^. Marres et al.^24^ observed that higher LNR in head and neck cancer could mostly be ascribed to LNs harvested at level V. Given that LNR was invariably relevant to the scope and extent of neck dissection, its predictable impact should be reconsidered. Unlike the volatile cutoff value of LNR, the dividing point of PLN appeared concentrated. Chan and Chan^13^ proposed a nodal staging system for oral cavity cancer with classification of patients into PLN ≤ 1 and PLN > 1 groups. In studies by Ricarte-Filho et al.^25^ and Park et al.^17^ on papillary thyroid carcinoma, an obvious distinction was observed while the threshold of PLN was set as 3. In the report of Feng et al.^21^ on oral and oropharyngeal cancer, the PLN > 2 subgroup exhibited markedly poorer outcomes, in keeping with our results. Thus, PLN number was adopted in our scoring system as it presents an explicit and consistent indicator with satisfactory predictive power. Notably, the number of histologically confirmed PLNs exceeded those presented in preoperative imaging tests in most cases, supporting the importance of making treatment decisions based on histological results.

Extranodal extension (ENE) has been increasingly recognized as a prognostic variable for many malignancies. The incidence of ENE was 38.6% in our study, which was markedly higher than that observed with MRI and color Doppler ultrasonography. We observed no correlation between ENE and number of metastatic LNs (*P* = 0.51). Moreover, the existence of ENE in isolated metastatic LN should not be overlooked. Chan et al.^26^ conducted a prospective study on recurrent NPC patient groups (without ENE treated with RND and with ENE treated with RND followed by brachytherapy). Microscopic ENE was detected in 25.9% of 158 patients. With inclusion of brachytherapy, the 5-year disease-free survival was still lower in the ENE (+) group, albeit to an insignificant extent. However, patients undergoing adjuvant chemotherapy and/or radiotherapy experienced poorer survival in our study, which may largely be explained by the selection bias of candidates for postoperative therapy. Consequently, the true efficacy of adjuvant therapy could not be verified. Such observations prompted prospective trials to provide multimodality treatment for high-risk patients owing to intrinsic higher malignancy and undesirable results. In view of the disparities in survival among recurrent NPC patients, it is imperative to improve survival in high-risk cases, which emphasizes the need to define the specific populations that should receive aggressive and effective interventions.

The EBV DNA value, which reflects tumor burden with high sensitivity, is universally considered a strong prognosticator for NPC. High EBV DNA levels have been associated with significant risk for poorer survival outcomes in many studies. In this cohort, owing to the strong correlation between the level of preoperative EBV DNA and lower neck involvement, the EBV DNA level failed to serve as an independent prognosticator in multivariable analysis. Moreover, its incorporation into the scoring model should be reconsidered as a clinical laboratory assay to evaluate extensive variations in EBV DNA levels among institutes. As reported by Kim et al.^27^, the sensitivity of EBV DNA quantitation ranged from 53% to 96%, highlighting the need to standardize laboratory procedures.

A significant limitation of this study was the absence of an external validation cohort from other hospitals. The role of the post-dissection EBV DNA test and dynamic changes before and after dissection are not discussed due to incomplete data on the whole cohort. Further prospective studies on high-risk regional recurrent NPC cases should be conducted and the effectiveness of multimodality treatment explored.

## Conclusions

Our study was conducted on a large regionally recurrent NPC cohort over the past 15 years. Distinct survival profiles among patients within this group were observed. Considering the limitations of conventional tumor variables and the AJCC rTNM system in predicting those at risk, we identified independent risk factors based on postoperative pathological results and established a preliminary robust scoring system to identify patients at high risk of regional recurrence. Our scoring model was accessible and practical and showed improved discriminatory ability relative to the rTNM staging system.

## Figures and Tables

**Table 1:** Demographics and characteristics of patients separated by PLN count

Variables	PLNs ≤ 2, *n* (%) (*n* = 211)	PLNs > 2, *n* (%) (*n* = 137)	*P*
Gender			
Male	152 (72.0)	113 (82.5)	0.029
Female	59 (28.0)	24 (17.5)	
Age (years)			
≤45	109 (51.7)	74 (54.0)	0.742
>45	102 (48.3)	63 (46.0)	
Time interval (from initial treatment to recurrence)			
Median (range)	21.0 (5.1–238.4)	18.9 (5.9–194.8)	–
Follow-up			
Median (range)	31.3 (4.1–198.2)	29.0 (4.1–167.8)	–
Recurrent N stage #			
N1	177 (83.9)	75 (54.7)	<0.001
N2	8 (3.8)	6 (4.4)	
N3	26 (12.3)	56 (23.6)	
Maximal diameter of LNs (mm)			
≤20	153 (72.5)	101 (73.7)	0.380
>20	58 (27.5)	36 (26.3)	
Bilaterality			
Unilateral	208 (98.6)	124 (90.5)	<0.001
Bilateral	3 (1.4)	13 (9.5)	
ENE			
ENE (−)	127 (60.2)	87 (63.5)	0.574
ENE (+)	84 (39.8)	50 (36.5)	
Lower neck involvement			
No	186 (88.2)	82 (59.9)	<0.001
Yes	25 (11.8)	55 (40.1)	
Surgical methods			
SND	153 (72.5)	89 (65.0)	0.153
RND	58 (27.5)	48 (35.0)	
Postoperative treatment			
No	166 (78.7)	86 (62.8)	0.002
Yes	45 (21.3)	51 (37.2)	
Presurgical EBV DNA value (copies/mL)*			
≤4000	81 (73.9)	96 (73.6)	1.000
>4000	93 (26.1)	75 (26.4)	

#Recurrent N stage was evaluated according to the AJCC TNM staging system (8th edition) for NPC. *Plasma EBV DNA test was not performed in patients treated before 2007. AJCC TNM, American Joint Committee on Cancer tumor-node-metastasis; EBV, Epstein–Barr virus; ENE, extranodal extension; PLN, positive lymph node; RND, radical neck dissection; SND, selective neck dissection.

**Table 2:** Univariable and multivariable analyses of prognostic factors for OS and PFS

Variables	Univariable		Multivariable	
	HR (95% CI)	*P*	HR (95% CI)	*P*
OS				
Gender	1.40 (0.78–2.52)	0.259	1.18 (0.65–2.15)	0.597
Age	1.61 (1.01–2.58)	0.048	1.53 (0.95–2.74)	0.083
LNs examined	0.91 (0.57–1.47)	0.701	§
PLNs	2.30 (1.44–3.69)	<0.001	2.19 (1.30–3.68)	0.003
LNR	1.72 (1.06–2.80)	0.029	§
ENE	2.55 (1.60–4.08)	<0.001	2.75 (1.69–4.47)	<0.001
Lower neck involvement	2.41 (1.48–3.93)	<0.001	1.78 (1.04–3.04)	0.034
Maximal diameter of LN	0.77 (0.45–1.32)	0.342	0.70 (0.41–1.21)	0.205
Bilaterality	0.87 (0.28–2.78)	0.820	0.46 (0.14–1.50)	0.201
PFS				
Gender	1.05 (0.72–1.54)	0.808	0.99 (0.67–1.47)	0.971
Age	1.03 (0.74–1.43)	0.868	1.00 (0.72–1.40)	0.995
LNs examined	0.93 (0.66–1.30)	0.652	§
PLNs	1.53 (1.10–2.14)	0.012	1.39 (0.96–2.00)	0.081
LNR	1.63 (1.16–2.29)	0.005	§
ENE	1.78 (1.28–2.48)	0.001	1.74 (1.24–2.46)	0.002
Lower neck involvement	2.03 (1.42–2.90)	<0.001	1.73 (1.16–2.57)	0.007
Maximal diameter of LN	0.89 (0.61–1.28)	0.524	0.83 (0.57–1.12)	0.315
Bilaterality	1.35 (0.66–2.75)	0.414	0.73 (0.34–1.57)	0.423

Categorical variables were gender (male *vs.* female), age (>45 *vs.* ≤45 years), LNs examined (>8 *vs.* ≤8), PLN no. (>2 *vs*. ≤2), LNR (>0.33 *vs*. ≤0.33), ENE (positive *vs.* negative), lower neck involvement (yes *vs.* no), maximal diameter of LN (>20 *vs*. ≤20 mm), and bilaterality (bilateral *vs.* unilateral). ^§^Variables were dropped from the model. *P* values were calculated with the Cox proportional hazards model using two-sided Wald test. CI, confidence interval; ENE, extranodal extension; HR, hazard ratio; OS, overall survival; PFS; progression-free survival; PLN, positive lymph node; RND, radical neck dissection; SND, selective neck dissection.

**Table 3:** Total points and estimated risk for OS and PFS with the novel scoring system

Groups	Total points	Incidence (%)	HR (95% CI)	*P*
OS				
Low risk	0	92.3	1 (reference)	–
Moderate risk	1	79.0	2.84 (1.34–6.00)	0.006
Moderate high risk	2	66.7	5.29 (2.43–11.51)	<0.001
High risk	3	55.6	9.69 (4.04–23.25)	<0.001
PFS								
Low risk	0	74.4	1 (reference)	–
Moderate risk	1	57.2	1.79 (1.15–2.78)	0.009
Moderate high risk	2	47.0	2.71 (1.66–4.43)	<0.001
High risk	3	37.0	3.79 (2.09–6.90)	<0.001

*P* values were calculated with the Cox proportional hazards model using 2-sided Wald test. HR, hazard ratio; OS, overall survival; PFS; progression-free survival.
