# Supplementary material for: Establishment of a prognostic scoring model for regional recurrent nasopharyngeal carcinoma after neck dissection
Source: Cancer Biol Med. 2020 Feb 15;17(1):227–36. doi: 10.20892/j.issn.2095-3941.2019.0263 (PMC7142849; doi:10.20892/j.issn.2095-3941.2019.0263)
Supplement: Supplementary file 1 [file cbm-17-227-s001.pdf]

Supplementary data

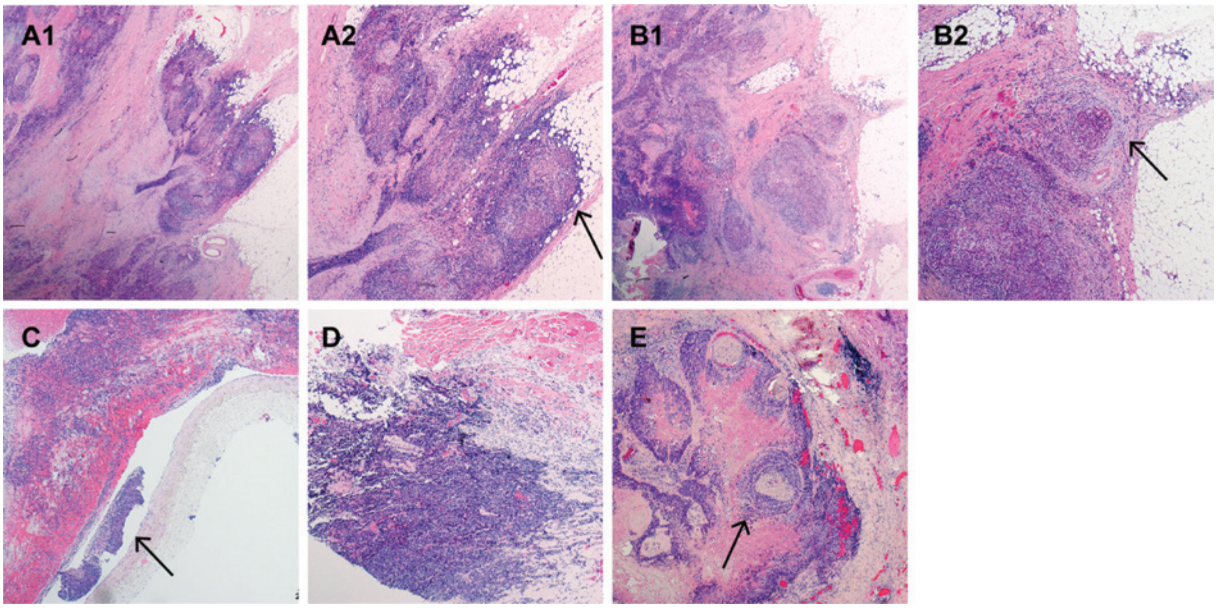

Figure S1 Several patterns of histopathologically confirmed extranodal extension (ENE). (A1–B2) Infiltration of metastatic foci through the capsule of lymph nodes (LNs) into surrounding fat tissue (arrow). (C) Cancer embolus in a blood vessel (arrow). (D) Infiltration of tumor tissues into striated muscle. (E) Infiltration of tumor tissues into a nerve bundle (arrow) (H&E staining, A1, B1, C–E: 40×; A2, B2: 100×).

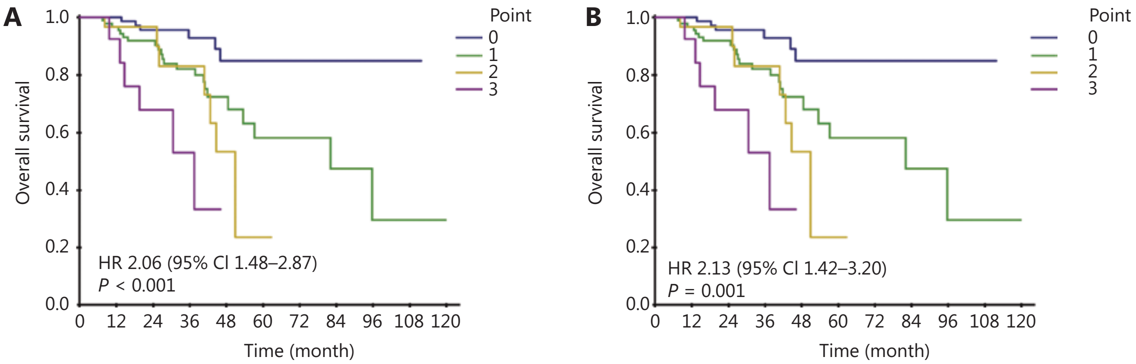

Figure S2 (A) Kaplan–Meier curves for overall survival (OS) in different risk groups based on the novel scoring model in patients receiving selective neck dissection (SND). (B) Kaplan–Meier curves for OS in different risk groups based on the novel scoring model in patients receiving radical neck dissection (RND) or modified radical neck dissection (MRND).
